# Community Capitals as Community Resilience to Climate Change: Conceptual Connections

**DOI:** 10.3390/ijerph13121211

**Published:** 2016-12-06

**Authors:** Shaikh Mohammad Kais, Md Saidul Islam

**Affiliations:** Division of Sociology, Nanyang Technological University, 14 Nanyang Drive, Singapore 637332, Singapore; shaikhmo001@ntu.edu.sg

**Keywords:** climate change, community resilience, community capital, environment, vulnerability

## Abstract

In the last few decades, disaster risk reduction programs and climate initiatives across the globe have focused largely on the intimate connections between vulnerability, recovery, adaptation, and coping mechanisms. Recent focus, however, is increasingly paid to community resilience. Community, placed at the intersection between the household and national levels of social organization, is crucial in addressing economic, social, or environmental disturbances disrupting human security. Resilience measures a community’s capability of bouncing back—restoring the original pre-disaster state, as well as bouncing forward—the capacity to cope with emerging post-disaster situations and changes. Both the ‘bouncing back’ and ‘moving forward’ properties of a community are shaped and reshaped by internal and external shocks such as climate threats, the community’s resilience dimensions, and the intensity of economic, social, and other community capitals. This article reviews (1) the concept of resilience in relation to climate change and vulnerability; and (2) emerging perspectives on community-level impacts of climate change, resilience dimensions, and community capitals. It argues that overall resilience of a place-based community is located at the intersection of the community’s resilience dimensions, community capitals, and the level of climate disruptions.

## 1. Introduction

Over the last few decades, community resilience, as one of the buzzwords of the time, has gained increasing academic and programmatic attention from social researchers, planners, community activists, and development practitioners [1,2,3,4]. Since disaster risk reduction has gradually moved away from a ’predict and prevent‘ model, in the context of specific hazards, to building community capabilities with regard to a wide range of rapid and slow onset shocks, stresses, and disruptions [5], resilience has now become one of the most desired paradigms for communities in addressing uncertainties, like oppressive political regimes, military invasions, global economic downturn, globalization threats, environmental disasters, and climate change disturbances [6]. Some authors even argue that the concept of resilience has become a global institution since it has now “emerged as a key driver of reform in disaster management and now underpins how governments and key organizations understand risk, uncertainty, and disaster” [6]. Although resilience is explored in a wide array of scholarly disciplines, including mathematics, physics, engineering, ecology, geography, economics, psychology, public health, sociology, anthropology, development studies, political science, and disaster management [7,8,9], the study of how resilience emerges in a climate-challenged community is scant. Researchers still face difficulties in dealing with the resilience of a community: at the conceptual level, defining and identifying resilient actions; at the operational level, modeling resilient behavior of communities in a single framework; and at the empirical level, collecting and analyzing community resilience data [10]. 

Adaptation and resilience literature has concentrated mostly on large-scale global, regional, national level scenarios of climate change and the recommended international-level policy options [11]. Since observed and future climate changes have, and will have, spatially- and socially-differentiated impacts experienced primarily at local levels, it is crucial to understand the community-level dynamics of climate disruptions [11,12,13]. Understanding how communities utilize their resources in effective ways to recover from the real and potential damage from climate threats is central to helping people plan for, and rebound from, environmental and climate disasters [7]. This article aims to contribute to the climate change and resilience literature through an in-depth and critical conceptual discussion of the exact mechanisms on how collective action and community capitals shape the degree of resilience of a geographically-bounded community facing both gradual and sudden changes in climate. Mapping the intersections between climate change, community resilience, and community capitals could provide essential information for policymakers in helping communities to avoid or withstand losses from climate hazards.

## 2. Climate Change Reality, Community Vulnerability, and Resilience

### 2.1. Climate Change: Reality of Our Time

Climate change is no longer a vague and indefinite future problem, but an unavoidable event that is damaging the planet at an alarming pace, an outcome of over 200 years of excessive greenhouse gas (GHG) emissions from fossil fuel combustion in energy generation, transport and industry, deforestation, and intensive agriculture [14,15]. For ecological, physical, and social systems and communities, climate change implications are intense and unsettling. It impacts human communities in many ways—the majority of which are compound, indirect, and sometimes ambiguous [16]—thus making scientific exploration of the total dynamics often problematic. 

Although climate change, through climate variability and extremes, has been a regular phenomenon throughout the history of the Earth, the current strain of global climate change is unique on two grounds—first, it is man-made, and secondly, it is happening more speedily than any time in the last fifty million years [17]. Since climate change is a stern global problem that will affect all of humankind within a very short period of time and has already affected several segments of the population in different regions with all its complexity, it is problematized on three main dimensions: climate change is highly complex, highly uncertain, and creates an environment of high decision stakes [18,19]. 

The scientific community is now overwhelmingly unanimous on the existence, causes, and impacts of global climate change. If we look at the cases of global warming, for example, we find that an unequivocal warming of the Earth’s climate system has been in operation since the Industrial Revolution began. Indeed, the last decade (2001–2010) was the warmest one in recorded history. Ever since the mid-1970s, the average global temperature has increased about 0.6 °C, almost 0.2 °C per decade [20]. The Fourth Assessment Report (AR4) of the Intergovernmental Panel on Climate Change (IPCC) predicts that world surface temperature is very likely to rise by 1.8 °C–3.4 °C by 2100, compared with 1980–2000 [20]. The IPCC in its latest Assessment Report (AR5) declared with 95% certainty that humans are responsible for more than half of the observed global warming between 1951 and 2010 [21]. Global fossil fuel burning is rising every year and if the trajectory continues, the Earth might become an unlivable planet by the year 2100 because of the consequences of rising global temperatures, which include rising sea levels, frequent heat waves, changed weather and seasonal patterns, frequent and prolonged droughts and floods, more tree deaths and insect damage, ocean acidification or a decrease in pH in ocean water, etc. From the discussion above, it is clear that climate change variability and extremes act as external stresses and perturbations to ecological and social systems and communities.

### 2.2. Defining Community

The notion of ‘community’ is fluid and is a social construct that needs to be defined on a case-by-case basis [22,23] focusing on the specific area of interest of investigation. A community can be viewed as “an affective unity of belonging and identity”, “a functional unit of production and exchange”, “a network of relations” among individuals, and “a unit of collective action” [24]. Communities are composed of compositional (i.e., characteristics of individuals living in an area), contextual (i.e., characteristics of the area where certain group of people live in), and functional (i.e., mechanisms and processes through which a community functions) components [22,24]. In community resilience studies, the term ‘community’ has been defined variously, including the totality of social system interactions, i.e., an affective unit of belonging and identity, and a network of relations, usually within a defined geographical space [25,26]; any group of individuals that share common interests, identify with one another, have a common culture, and participate in shared activities [27,28]; a group of people living within the territorial boundaries of an administrative unit [29]; and a place-bound neighborhood as well as an interest or kinship-based social unit [30]. A community’s most significant feature, in the context of resilience to climate changes, is its capacity “to collectively identify problems, take decisions and act on them and to allocate resources” [31]. Although it is possible to view communities in various levels of size and length from a tiny area of a village to a global community, generally, social resilience researchers locate community at an intermediary level in terms of a nested hierarchy of geographical scale, in which local community is placed above household level and below regional level [26]. 

In this study, community is defined as a place-based geographical entity, located at the intersection of household and regional levels, which displays few distinct functional characteristics of its members that include (1) community members interacting on a somewhat regular basis; (2) this interaction is not significantly mediated by the state; and (3) members have some degree of shared preferences or beliefs [32]. We base our analysis of community resilience on this conception of community. 

### 2.3. Community Vulnerability and Resilience

A community’s climate risks and coping capacities can be assessed through two essential approaches: social vulnerability and resilience [7]. Vulnerability of a system, primarily from an ecological point of view, is composed of three components: (1) exposure to perturbation—the degree to which a system is in contact with the perturbation; (2) sensitivity to perturbation—the degree to which a system is affected by a disturbance; and (3) capacity to adapt—a system’s ability to adjust to a disturbance [33]. An intricate range of economic, political, social, and physical factors jointly determines the vulnerability of a person or community. Scholars have differentiated between human and non-human components and distinguished between human, social, and physical vulnerabilities [34]. Thus, vulnerability is the measure of a system’s fragility with regard to environmental and other threats. Only a resilient community (more in the subsequent sections) can successfully overcome stresses including climate disruptions. Resilience is the long-term capacity of a system to deal with change and continue to develop [35]. Resilience to climate change of a community can be defined as a combination of resistance to frequent and severe disturbances, capacity for recovery and self-organization, and the ability to adapt to new conditions [36]. 

Recently, social scientists have examined the relationship between vulnerability and resilience of a system or community with regard to environmental and social disturbances [7,12,13]. We can discern at least three broad streams of the relationships between the two sister concepts: (1) the two are opposite to each other [7,22,25,26,37,38]; (2) one is a subset of the other [33,39]; and (3) the two are overlapping, but separate, concepts [7,13,34,40,41,42]. 

The bottom-line of the arguments is that while both approaches can be applied to rapid and gradual hazards and changes, the vulnerability framework is more appropriate for addressing rapid onset events, like cyclones or earthquakes, whereas the resilience perspective is more suitable for addressing slow onset transformations like drought, famine, long-term temperature shifts, and sea-level rise. Similarly, both concepts are overlapping in the sense that although a community can possess characteristics that can determine only its vulnerability or only its resilience, it can have multiple socio-economic characteristics that influence both its vulnerability and resilience [42]. Moreover, central to vulnerability-reducing programs is a top-down manner of service delivery from state organizations to local communities, whereas at the heart of resilience initiatives are the “bottom-up view of individual, organizational, and community capability and capacity” [6]. Thus, resilience, compared to vulnerability, has great potential to bring together cumulative, progressive, and dynamic knowledge, insights and works in dealing with and learning from hazards and threats.

Cannon and Muller-Mahn [43] argue, opposing others, that a paradigm shift from a vulnerability to a resilience perspective is dangerous. According to them, “vulnerability involves a clear, economically and politically induced condition that theorizes the way that people are exposed to a lesser or greater degree of risk” [43]; while resilience, derived from natural or technological sciences and with a focus on systems (or ecosystems) approach, fails to grapple with human society’s socio-economic components. Despite few limitations of the concept of resilience—as delineated by Cannon and Muller-Mahn [43], for example—academic and policy attentions are now shifting from a vulnerability approach to a resilience perspective because the latter is viewed as “more proactive and positive expression of community engagement” [42], especially with regard to slow onset hazard reduction and climate adaptation. The section below focuses more on this perspective. 

## 3. Resilience: The New Frontier

### 3.1. Conceptualizing Resilience

Resilience is popularly understood as the degree of elasticity in a system, its ability to rebound or bounce back after experiencing some stress or shock. The concept of resilience is unusually “plastic” [44], “highly ambiguous” [45], vague and malleable [37], and often used as a promiscuous approach, like global commodity chains [46,47] incorporating different meanings in different contexts. This is because of a blending of descriptive (what the case is) and normative aspects (what the case should be) within its conceptualization [37]. Although there are scholarly arguments on the positives and negatives of the ambiguity and plasticity of the concept of resilience [34,37,44,45,48], the positives and the negatives of the malleability of the concept can be offset through a ‘division of labor’ of the resilience scientists. Resilience as a descriptive concept, with precise definition and meaning and operationalized quantitatively, can be applied to hard systems in physical, ecological, and climate hard sciences. Resilience, in this usage, can be dubbed as ecosystem resilience, ecological resilience, engineering resilience, or simply, resilience. Resilience as a normative concept, on the other hand, with inherent vagueness and malleability, and with an opportunity to be operationalized qualitatively, can be applied to trans-disciplinary analyses of soft systems like social, political, and cultural systems. In this usage, resilience can be termed as ‘social resilience’, ‘cultural resilience’, ‘economic resilience’, or ‘community resilience’.

In its basic form, the meaning of resilience incorporates the idea of self-organization after experiencing a shock. Ecologists and social scientists conceptualized ‘resilience’ in various ways. The resilience process of a system has two discrete elements: a significant amount of disturbance that can destroy or disrupt the system, and the system’s resilience capacity to withstand, adapt to, or recover from the traumatic event [49,50].

Resilience of a system (or community) incorporates both static and dynamic properties. On the one hand, resilience is conceptualized as an inherent or antecedent condition or state or ‘outcome’ [10,42,45,51]. Inherent resilience reflects an ontological definition of resilience which focuses on the system’s ‘being’ [52,53], i.e., its ability to function well under normal circumstances in non-crisis periods and its ability to reinstate the pre-crisis stability (i.e., bounce back) if any catastrophe strikes it. In other words, inherent resilience can be thought of “as a snapshot in time or as a static state” [42], a system’s latent attributes [49,50]. On the other hand, a system’s resilience is also its adaptive quality during and after a crisis—it is a ‘process’. This dimension is related with the phenomenological view which focuses on ’becoming’ [52] or ’doing’ [53]. In this view, resilience is not a fixed property of the system; rather, it incorporates the idea that a resilient system has flexibility in responding to hazards in that the system’s initial structure or function might undergo some necessary changes—resilience is viewed as “a quality, characteristic or result that is generated or developed by the processes that foster or promote it” [34]. Dovers and Handmer view outcome vs. process dimensions of resilience as reactive and proactive resilience. At the center of reactive resilience is the “quest for constancy or stability” [54], while proactive resilience “accepts the inevitability of change and tries to create a system that is capable of adapting to new conditions and imperatives” [55]. Static and dynamic resilience can lead to diverse community resilience programs. For example, outcome-oriented resilience schemes tend to espouse ‘command and control styles’ which aim to retain the social status quo. Process-oriented disaster resilience schemes, on the other hand, focus on the series of actions that enhance a community’s coping capacity over time.

### 3.2. Evolution of the Concept of Resilience in Academic Discourses

The concept of resilience has its origin in the sciences of mathematics and physics [9,45,56,57], and ecology [58,59]. In its original usage, the concept describes the ability of an object or system “to return to equilibrium after a displacement” [9]. The English word ‘resilience’ has its origin in Latin ‘resiliens’ and its derivatives ‘resilio’ and ‘resilere’ [51]. In essence, resilience translates to ‘recoil’ [6] or ‘to jump back’ [34,60,61]. The earliest English usage denoted the repercussive qualities of sound, namely ‘resilience of echoes’ [6]. Later, the term was used in engineering science to mean the obdurate quality of materials. In the twentieth century ecologists grabbed the concept and during the last half century it has been in use in various disciplines. Thus, ‘resilience’ has now, in its ‘third wave’ [56], become a hot issue in sociology and other social sciences. Within social and behavioral sciences, developmental psychology was the first discipline in which the study of resilience evolved [49,50,62,63]. Currently, sociologists are contributing notably in expanding the concept of resilience at social levels.

Wilson [25,26] conceptualized the evolution of the resilience concept in the social sciences in three phases over the last five decades. ‘Ecological resilience’ was the focused notion during the late 1960s and early 1970s with an emphasis on descriptive and non-normative studies on how ecosystems responded to disturbances. Later on, the resilience approach diffused in various social science studies taking an extended ecological resilience definition. During the late 1970s through 1990s, social scientists deployed the resilience concept in ‘social-ecological systems’ study. In this phase, the notion of resilience emerged as a ‘boundary object’ between the natural and social sciences [26,37]. Resilience was defined in this stream of research as “the capacity of a system to absorb disturbance and reorganize so as to retain essentially the same function, structure, and feedbacks—to have the same identity” [64]. The conception of ‘social-ecological resilience’ was still burdened by its substantial reliance on “deterministic and positivist natural science-based behavioral assumptions” [26] which is not always applicable to human communities and systems. Social scientists began to feel discomfort in using this framework for social systems because a social system incorporates structural, as well as agency, variables within it, while an ecological system is primarily built on structural components. Thus, the concept of ‘social-ecological system resilience’ is incapable of conceptualizing the totality of the role of human agency factors in the resilience of a social system. 

In order to overcome the inadequacy of ‘social-ecological resilience’, social scientists, after the 1990s, began to use the third strand of research focusing solely on human groups and communities by deploying the new concept of ‘social resilience’ [26]. Social resilience has been defined as the ability of human communities to withstand external shocks to their social infrastructure, such as environmental variability or social, economic, and political upheaval [58,59]. Traditionally, resilience theorists did not focus on power relations, politics and culture; instead, their emphasis was on the structure and function of a system. Social resilient theorists brought about the question of policy, political, social, economic, psychological, and ethical parameters into the resilience spectrum [25,26,58,65]. Social scientists now engage a ‘bottom-up’ approach in comprehending human drivers and indicators at the community level, in which human–environment interactions are only one of many constituents. One of the differences between ecological resilience and social resilience is that while the former is linear the latter is non-linear, in the sense that due to social learning processes and social memory social systems can never go back to their original status after a blow (but ecosystems may be able to do that). For a human community, social resilience can be both preventive to shocks through avoiding poor outcomes by developing adaptive mechanisms and curative for recovery after a traumatic or catastrophic disturbance [26]. Through its anticipatory nature, a social community may develop human adjustment processes that push the after-shock system to a new, and sometimes better, state—hence, at the heart of social resilience is ‘bouncing forward’ or ‘move on’, not ‘bouncing back’ [41,66]. Thus, the goal for adaptive capacity after a disruption would often not be to attempt to restore the original state but to “use learning processes and social memory as a basis for the creation of a qualitatively different more resilient community” [26]. So, in this view, social resilience is both an outcome, meaning an upgraded coping ability of communities, and a process, referring to community-level dynamic changes and learning to withstand shocks over time and to take responsibility and control of their development trajectories [24,25,26,67]. 

### 3.3. Typology of Resilience

Since the concept of resilience has different connotations in different contexts, it can be categorized in multiple ways. In order to comprehend the complex dynamics of resilience, we can classify resilience based on the nature of each of its three components: threat, system, and response. 

#### 3.3.1. Nature of the Threat

Based on the nature of the disturbance a system or a particular aspect of a system focuses on, Folke and colleagues distinguished between specified and general resilience [38]. When a system develops coping mechanisms with regard to a specific threat, its adaptive capability is termed as specified resilience. For example, if a climate-challenged human community develops resilience in addressing the problem of salinity intrusion—but does not become resilient to drought, flood, cyclones, and other climate threats—its resilience can be termed as specified resilience. Specified resilience answers the question: “resilience of what to what?” [68]. Generally, the majority of the adaptation programs for communities designed by governmental and non-governmental organizations in developing countries focus on specific problems and issues which, in turn, result in developing sector-wise resilience. However, too much focus on a specific event or sectors can possibly lead to new kinds of instability of the system in facing a multitude of challenges. Thus, specified resilience is not holistic in nature. General resilience, on the other hand, is the resilience which a system grows in order to address all sorts of shocks simultaneously. This type of resilience program addresses all of the potential threats that affect the system as a whole or part of it. Thus, general resilience is holistic. Similarly, Begon and colleagues, using the concept of stability synonymously with the current usage of resilience, distinguished between local stability and global stability [55,69]. Local stability means the tendency of a community to reinstate its original state ‘when subjected to small perturbation’ [69]; global stability is the capability of a system to return to its original state after comparatively large disturbances. 

#### 3.3.2. Nature of the System

**Figure 1:**
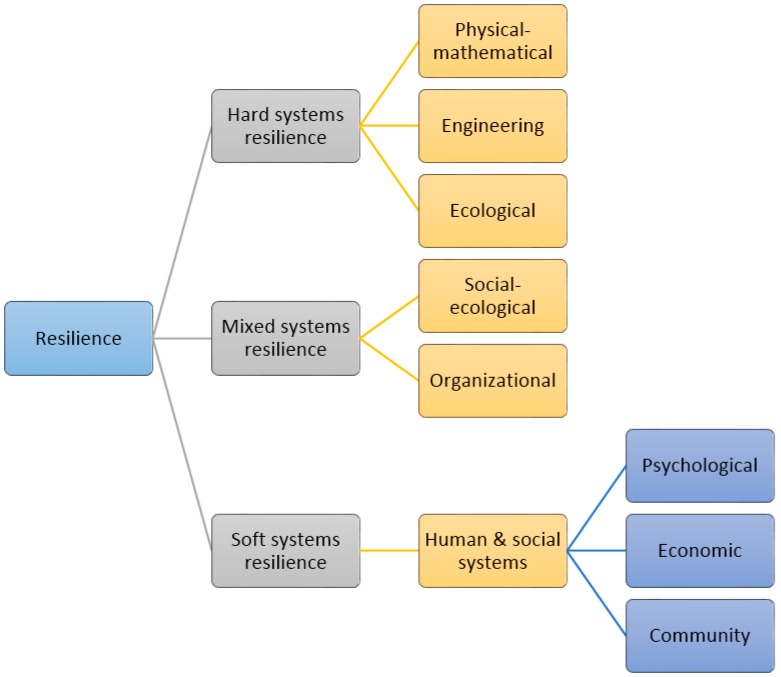
Major types of resilience (based on the nature of the system).

In terms of the nature of the system, resilience can be categorized in a number of ways. Following Checkland and Scholes [70] and Handmer and Dovers [55], we can distinguish between resilience of hard, soft, and mixed systems (Figure 1). As mentioned earlier, resilience of hard systems can be characterized as descriptive, non-normative [37], tangible, precisely defined, and quantitatively measurable [55]. The major systems that have this type of resilience include physical–mathematical, engineering, and ecological systems. In physical and mathematical sciences, resilience is viewed as a part of stability analysis and synonymously with elasticity. Resilience of a material or system is linked with its dynamic behavior to return to the original point of equilibrium. Resilience can be defined as the speed with which a system returns to equilibrium after displacement, irrespective of the number of oscillations required [56]. The more a system is resilient, the more displacement force is required to destabilize it. Structural and engineering science borrows the original meaning of resilience from physical–mathematical sciences. Engineering resilience is focused on “efficiency, control, constancy, and predictability—all attributes at the core of desires for fail-safe design and optimal performance” [71]. In one of its applied levels, engineering resilience, as the concept of seismic resilience of buildings, denotes the following properties of the system: “reduced failure probabilities; reduced consequences from failures, in terms of lives lost, damage, and negative economic and social consequences; and reduced time to recovery” [72]. While both physical–mathematical and engineering resilience emphasize on a system’s capacity to return to the original state, ecological or ecosystem resilience focuses on the system’s ability to absorb changes. Ecological resilience can be defined as the buffer capacity of ecosystems to absorb externally caused perturbation, maintain robust functions, and survive systemic shocks [6,73]. In this approach, ecosystems are viewed as constantly changing systems and entities. Thus, maintaining ecosystems functions is a dynamic process of renewal and reorganization. In this sense, ecological resilience shifts from the original mathematical-engineering idea of single equilibrium to multiple points of stability of a system [45]. 

Contrary to resilience of hard systems, resilience of soft systems can be characterized as vague and malleable, normative, intangible, and hard to be quantitatively measured. The major forms of resilience of human and social systems that fall under this category include psychological, economic, and community resilience. Psychological resilience, developed independently of mathematical or ecological origins, framed resilience as an adaptive capacity of humans. In early psychiatric literature, children who could withstand high-risk circumstances were termed as ‘invulnerable’—and eventually ‘invulnerability’ was replaced by ‘resilience’ in psychology texts [6,45,74]. Humanistic psychology defines resilience as an individual’s capacity to thrive and fulfill potential despite or perhaps even because of such stressors resilient individuals seem not only to cope well with unusual strains and stressors but actually to experience such challenges as learning and development opportunities [75]. Economic resilience refers to the incorporation of the resilience approach into economic activities including “production inputs and outputs, demand- and supply side effects, inherent and adaptive abilities, and levels of the economy” [76]. Contrary to pre-disaster mitigation efforts, Rose [10,51] defines resilience based on ‘post-disaster conditions and response’. Rose also puts forward that resilience in economic sectors can take place at three levels: “microeconomic—individual behavior of firms, households, or organizations; meso-economic—economic sector, individual market, or cooperative group; and macroeconomic—all individual units and markets combined, including interactive effects” [51]. The concept of community resilience was first used perhaps by Judith Kulig and colleagues working in health promotion services in Alberta, Canada [45,77]. Community resilience refers to the capacities and capabilities of a human community to ‘prevent, withstand, or mitigate’ any traumatic event [49,50]. Though the challenges to a community are thought of as coming from external stresses like social, political, or environmental changes [58], the threats may also be the result of a community’s internal evolutionary processes of vulnerability [26,67,78]. 

In between the hard and the soft systems there is a third category, which we can tag as ‘mixed systems’. Mixed systems resilience is a hybrid concept—a boundary object in which descriptive and normative connotations are intermingled [37,26]. This type of resilience lies at the border between natural and social sciences. We can cite social–ecological resilience and organizational resilience as two examples of mixed systems resilience. The social–ecological systems (SES) approach treats humans as part of ecosystems—the nature–culture divide is viewed as arbitrary and artificial. Adger and colleagues [12,58] defined social–ecological resilience as the capacity of linked social–ecological systems to absorb recurrent disturbances so as to retain essential structures, processes, and feedbacks. Organizational resilience of business enterprises is another example of mixed system resilience. As an area of ‘corporate strategy’ [79], enterprise (or, organizational) resilience incorporates the ideas of risk management and governance processes: enterprise resilience marries risk assessment, information reporting, and governance processes with strategic and business planning to create an enterprise-wide early warning capability that is embedded in the business of the company [80]. Thus, enterprise resilience puts equal weights on physical-structural aspects and company culture—a combination of both hard and soft systems approaches. 

#### 3.3.3. Nature of the Response

Based on the nature of the response that a system generates to hazards or disturbances, Handmer and Dovers [55] suggested a three-way generic classification of resilience: resistance and maintenance; change at the margins; and openness and adaptability. The type 1 resilience, resistance and maintenance, is the most basic form. A social system of this type tries to avoid change, denies the existence of any problem, allocates resources in order to maintain the status quo, and does not question any underlying assumptions or power asymmetries in society. This approach is favored by the elites of society who fear the loss of their authority if any drastic change occurs in the society. Since this type of resilience lacks flexibility, a system containing it cannot adjust to new situations. Having this form of resilience, a system would collapse instead of transform. The type 2 resilience, change at the margins, is the standard approach to address disturbances. A system with this type of resilience admits the existence of a problem; acknowledges its repercussions; acknowledges the non-sustainability of the current system; and advocates reforms in a degree which does not challenge the societal bases or governance regimes. Thus, this approach, dominant in the West, addresses symptoms, not the underlying causes [55]. Type 3 resilience, openness and adaptability, is the deepest form of resilience which directly addresses the fundamental causes of hazards; and advocates major political and cultural shifts through transformation in over-arching political economy regimes and associated cultural discourses on development, risk, and security. 

The above discussion on various approaches to resilience serves as the theoretical base of this study. Since we focus on community resilience, it is now clear that community resilience is soft, social, and one of the most adopted approaches in addressing disasters in societies in recent time. The central strength of a community resilience approach, as a type of social resilience, is its characteristic both as an ‘outcome’ and a ‘process’. Since identifying community resilience is the key purpose of this paper, the following section briefly outlines the major relevant issues. 

## 4. Community Resilience: The Focused Level

Every level of human institutions or organizations, from the individual/household to the global, shows resilience characteristics with varying dynamics. In its multi-scalar dimension, resilience is more direct and tangible in the individual/household level, and gradually takes on a more abstract and intangible form at larger spheres [25,26]. Thus, it is imperative for studies to clearly define and characterize resilience, the focused social level, and measuring methods. For example, as a global problem, climate change decision-making and initiatives come from the larger global, regional, or national strata, but are tangibly implemented at household or community levels. Communities are particularly important in conceptualizing social resilience because this level is at the intersection of micro-individual-household and macro-national-global levels, which can be downscaled or up-scaled [26,42]. In this section, we will focus on the characteristics of a climate resilient community, and discuss how community capitals create resilience to climate change. 

### 4.1. Characterizing a Climate Resilient Community

Community resilience, or ‘regional resilience’ [81] has been defined by social scientists in various ways, including “those features of a community that in general promote the safety of its residents and serve as a specific buffer against injury and violence risks, and more generally, adversity” [49]; a community’s ability to maintain, renew, or reorganize social system functions and ecological functions [50]; “a process linking a set of networked adaptive capacities to a positive trajectory of functioning and adaptation in constituent populations after a disturbance” [9]; “the existence, development, and engagement of community resources by community members to thrive in an environment characterized by change, uncertainty, unpredictability, and surprise” [82]; and “(a community’s) social capital, physical infrastructure, and culturally embedded patterns of interdependence that give it the potential to recover from dramatic change, sustain its adaptability, and support new growth that integrates the lessons learned during a time of crisis” [28]. 

In the literatures on community resilience [2,5,9,28,83,84], we discern the following characteristics of a resilient or ‘viable’ [48] community, especially in terms of both rapid and slow onset socio-economic, environmental and climate changes. A resilient community:

- Takes intentional action to enhance the personal and collective capacity of its members and institutions to respond to, and influence the course of social and economic change.
- Is organized. It has the capacity to recognize problems, institute priorities, and act.
- Fosters the factors that enhance community resilience by improving community members’ capabilities; i.e., by learning to live with change and uncertainty; nurturing diversity for reorganization and renewal; combining different kinds of knowledge; and creating opportunity for self-organization.
- Adapts to constant changes. It does not treat shocks and disturbances only as episodic, but regards many of them as constant and gradual threats.
- Builds its resilience through cumulative mechanisms and pathways over time. It is knowledgeable and skillful in assessing, managing, and monitoring its risks. It can learn new skills and build on past experiences.
- Is multi-scalar; it acts at the individual, community, and regional levels, deploying its internal as well externally-networked resources in tackling and coping to adversaries.
- Assists its members to navigate to resources as well as to negotiate for the resources they need.
- Is relatively autonomous and self-sufficient in relation to economic decision-making. It has wider economic diversities with a broader range of employment options, income, and financial services (economic capital). It is flexible, resourceful, and has the capacity to accept uncertainty and respond proactively to change.
- Is rich in community capitals including economic, social, built, political, and environmental capitals.
- Is capable of clearly identifying its barriers (i.e., pre-disaster vulnerabilities, social class, mistrust, race and ethnicity, gender) and facilitators (i.e., access to community resources, local community civic and faith-based groups, and bonding–bridging–linking social capitals).
- Is connected to external actors (including family friends, religious groups, and government) who deliver a wider supportive environment and supply goods and services when needed (linking social capital).
- Has physical infrastructures and services (built capital) that include resilient housing, transport, and power, water, and sanitation systems. It has the ability to retain, repair, and renovate them.
- Can manage its natural assets (environmental capital). It recognizes their value and has the ability to protect, enhance, and maintain them.

### 4.2. How Community Capitals Create Resilience to Climate Change

From the above discussion it is clear that a climate resilient community has sufficient assets and resources that facilitate its coping capacity with long-term changes. A balanced combination of various ‘community capitals’ [82]—sometimes variously termed as community resources [48,53], community energy [48], or community capacities [53]—enhances resilience of a community to disturbances including climate change disruptions. Community capitals are resources of a community that are invested for the collective wellbeing of the entire community. Social scientists frequently mention human capital (i.e., an individual’s innate and acquired personal attributes, such as work skill, education, knowledge, and health, which contribute to the ability to earn a living and strengthening the community), cultural capital (i.e., community’s worldview, values, and norms), financial or economic capital (i.e., material property, wealth, and other financial sources available to be invested for business development, civic, and social enterprises), physical or built capital (i.e., physical infrastructure of a community including machinery, homes, factories, water, roads, transport, shelter, and energy), political capital (i.e., community members’ access to resources, power, and power brokers), environmental or natural capital (i.e., availability and sustainable use of natural resources for human consumption), and social capital (i.e., the extent of social networks) [9,22,26,28,30,53,82,84,85]. Social capital is an ‘umbrella term’ [25,26] that can incorporate cultural and political capitals of a community. Social capital may further be divided into bonding capital (i.e., close ‘inward looking’ horizontal ties of social network that build cohesion within a community), bridging capital (referring to loose horizontal ties of ‘outward looking’ social networks across various social and ethnic groups), and linking capital (referring to vertical relationships across power or authority gradients) [82,85]. 

According to Wilson [26], a community’s resilience can be conceptualized on the basis of how well the ‘critical triangle’ of three major community capitals—economic, social, and environmental capital—are developed in a certain community and how these capitals interact. A community is strongly resilient when all three capitals are well developed in it, while it is weakly resilient when only one or no capital is well developed in it, and furthermore, a community is moderately resilient when two capitals are well developed. 

As noted in an earlier section, the IPCC [14] points out a number of climate-related drivers that affect the global environment. The drivers include warming trends, extreme temperatures, drying trends, extreme precipitation, damaging cyclones, flooding, storm surges, ocean acidification, temporal shifts in seasonality, and resulting sea-level rise (SLR) and salinization of water and soil. All of these ‘signatures of climate change’ [86] have devastating repercussions on human livelihoods and communities. Human communities are negatively impacted by recent global warming in numerous ways, such as negative impacts on average crop yields and increases in yield variability; urban risks associated with water supply, housing, and energy systems; decreased access to water for poor people due to water shortage and growing competition for water; displacement, and involuntary and forced migration associated with extreme events; violent conflict arising from deterioration in resource-dependent livelihoods such as agriculture and pastoralism; declining work productivity, increasing morbidity (i.e., dehydration, heat stroke, and heat exhaustion), and mortality from exposure to heat waves. Particular segments of people, including homeless people, children, construction workers, and women, are more vulnerable to increasing morbidity and mortality [14]. All of these current and projected consequences of climate change on human livelihoods have, in turn, negative impacts on overall community capitals—economic, social, physical, natural, and others. A community’s resilience to climate change depends on how the community withstands, and recovers from, climate shocks through its economic, social, and other community capitals. 

However, community capitals are neither equally distributed among community members nor are they available to a community as group—often some capitals are under the capacity of particular individuals or groups of individuals. For example, human capital varies from individual to individual, and economic and political capitals may vary from class to class. Nevertheless, all of the capitals available to a community can mutually support and enhance each other if they are utilized optimally by the community members. Climate events can have variegated impacts on geographically-bounded communities. They can negatively impact one form of capital but can facilitate other forms. For example, sudden changes like floods can destroy physical infrastructures in a community but they can improve natural capital and are likely to attract a financial influx through governmental, and other, rebuilding and rehabilitation schemes. On the contrary, slow onset changes, such as sea-level rise or drought, can destroy financial and environmental capital of a locality, but do not attract support from outside. 

As we noted earlier, a resilient community has distinct features with regard to how it utilizes its resources in addressing climate shocks. A community’s resilience depends on how rich the community is in terms of various capitals; how quickly and how well it recovers from the losses through effective use of resources and capitals; how the whole community works as a unified team; how committed and persevering its members are; how well it identifies its barriers and facilitators; how well it is horizontally and vertically connected to other groups and institutions, and how well it uses these connections; and how dynamic and strategic are its community leaders. We can term all of the above characteristics of a community as its resilience dimensions [53]. 

**Figure 2:**
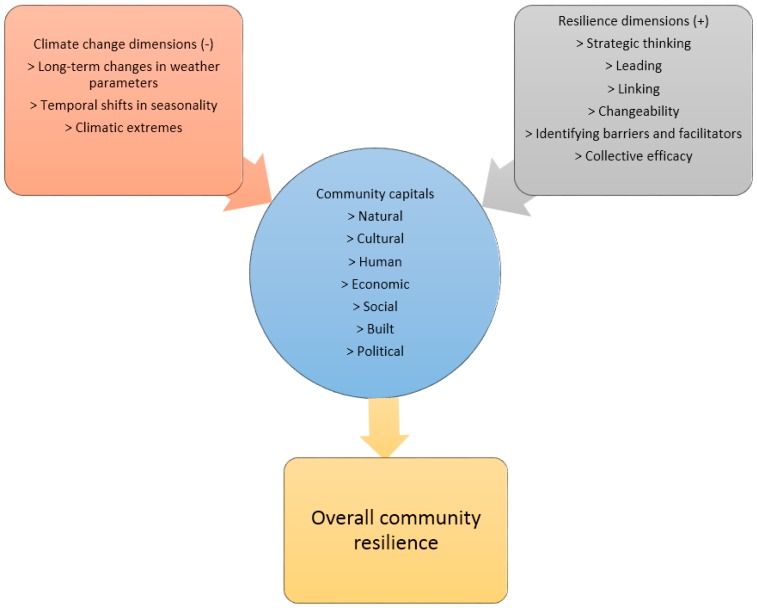
A conceptual model for climate change, community resilience and community capitals.

Thus, it can be concluded that although climate change phenomena can have indirect positive consequences on social, economic, and other community capitals, generally, climate variability and extremes have immediate and direct negative impacts on community resources through destabilizing or destroying them. Community resilience dimensions, on the other hand, have short-term and long-term direct positive influences on community resources and capitals through recovering and reinstating community strength. Community capitals serve as the working ground for both climate change impacts and resilience dimensions—either positively or negatively. Figure 2, adapted from McCrea et al. [53], presents schematic relations between climate change impacts, community capitals, and community resilience. Though this sketch does not imply a rigid flow of events or outcomes, it depicts the main argument of this paper that, in a human community, climate change dimensions have overall negative impacts on community capitals and resilience dimensions have positive impacts. The net result of the impacts of these two dimensions on community capitals is indicative of the extent of community resilience to climate change. 

## 5. Conclusions

Climate change generates risk to human society in a multitude of intermingled ways, including long-lasting changes in mean temperatures, precipitation, and other climate parameters, as well as secondary effects, such as sea-level rise, temporal shifts in seasonality culminating into extreme and abnormal conditions at local levels, and increased climate extremes that can elicit floods, cyclones, fires and other natural disasters. Resilience to local impacts of global climate change requires sensitivity to the local milieu since other social, cultural, economic, political, ethnic, and environmental risks and opportunities define context-specific human well-being [87]. Community-level resilience is created and re-created through constant organization, disorganization, and reorganization of resources and capitals of the community. This article contributes to disaster and resilience studies by clearly establishing the conceptual connections between community level impacts of climate change, community capitals, and community resilience.
